# Light and Capillary Waves Propagation in Water Fibers

**DOI:** 10.1038/s41598-017-16906-0

**Published:** 2017-11-30

**Authors:** Mark L. Douvidzon, Shai Maayani, Leopoldo L. Martin, Tal Carmon

**Affiliations:** 1 Technion, Department of Nanoscience and Nanotechnology RBNI, Haifa, Israel; 2 Technion, Department of Mechanical Engineering, Haifa, Israel

## Abstract

The confinement of light and sound, while they are traveling in fibers, enables a variety of light-matter interactions. Therefore, it is natural to ask if fibers can also host capillary waves. Capillary waves are similar to those we see when throwing a stone into a puddle. Such capillary waves are prohibited in microfluidic devices where the liquid is bounded by solid walls. In contrast, we have fabricated fibers, which are made entirely from water and are suspended in air. The water fiber can therefore move, e.g. in a resonant mode that reassembles the motion of a guitar string. In our experiment, light guided through the water fiber allows optical interrogation of is capillary oscillations. Co-confining two important oscillations in nature: capillary and electromagnetic, might allow a new type of devices called Micro-Electro-Capillary-Systems [MECS]. The softness of MECS is a million times higher when compared to what the current solid-based technology permits, which accordingly improves MECS response to minute forces such as small changes in acceleration. Additionally, MECS might allow new ways to optically interrogate viscosity and surface tension, as well as their changes caused by introducing an analyte into the system.

## Introduction

Fibers constitute the backbone of modern communication^1^. They are used in laser surgery^2^ as well as in the generation of coherent X-ray^3,4^, guided-sound^5^ and supercontinuum^6^. We fabricate optofluidic^7–9^ fibers^10–13^ and use them to co-host light and capillary oscillations. Our water fibers are made by water bridging^14–21^ an optical tapered-coupler^22^ to a µlensed coupler^23^. Our water-fiber diameter is at the tens-of-micron scale. At this scale, cohesive forces between intimate particles of liquid result in tension minimizing the liquid surface. This type of restoring force supports capillary waves, which propagate on the liquid phase boundary. Capillary waves are unique to liquids, in contrast to light and sound, which are common to all phases of matter. Capillary oscillations were widely studied in flat interfaces^24–27^ and in droplets^28–30^, which makes it natural to extend such opto-capillary effects to fibers. Previous studies in liquid waveguides^10–13^ were using liquid-core, solid clad waveguides. If we take such a waveguide and change its core to a runny (low viscosity) liquid and its cladding to air, the liquid phase boundary will be able to move freely. Our water fibers^31^ operate in the underdamped regime (quality factor >1/2) where a capillary wave can propagate a distance longer than a wavelength. Fabricating a suspended water string creates some structural hurdles. To mitigate these structural challenges and to build a floating water bridge, we use an electric-field based technique reported in 1893. In this report^14^, a voltage difference between two beakers resulted in an unsupported water thread that emerged while fluidically connecting the two reservoirs. Two mechanisms participate in carrying the weight of the water fiber. The first is surface tension and the second is the electric field, also referred to as dielectric force^19–21^. Such water bridges were demonstrated at lengths longer than 3 centimeter^32^ or with a diameter thinner than 20 nano-meters^16^. Yet, water bridges were never used for guiding light. These types of water threads can, in principle, guide electromagnetic-, acoustical- and capillary-waves, which make them attractive for sensors and labs-on-chips^7–9^, where several different waves can interact. This is in contrast to multi-spectral systems where different frequencies of the same wave participate.

**Figure 1:**
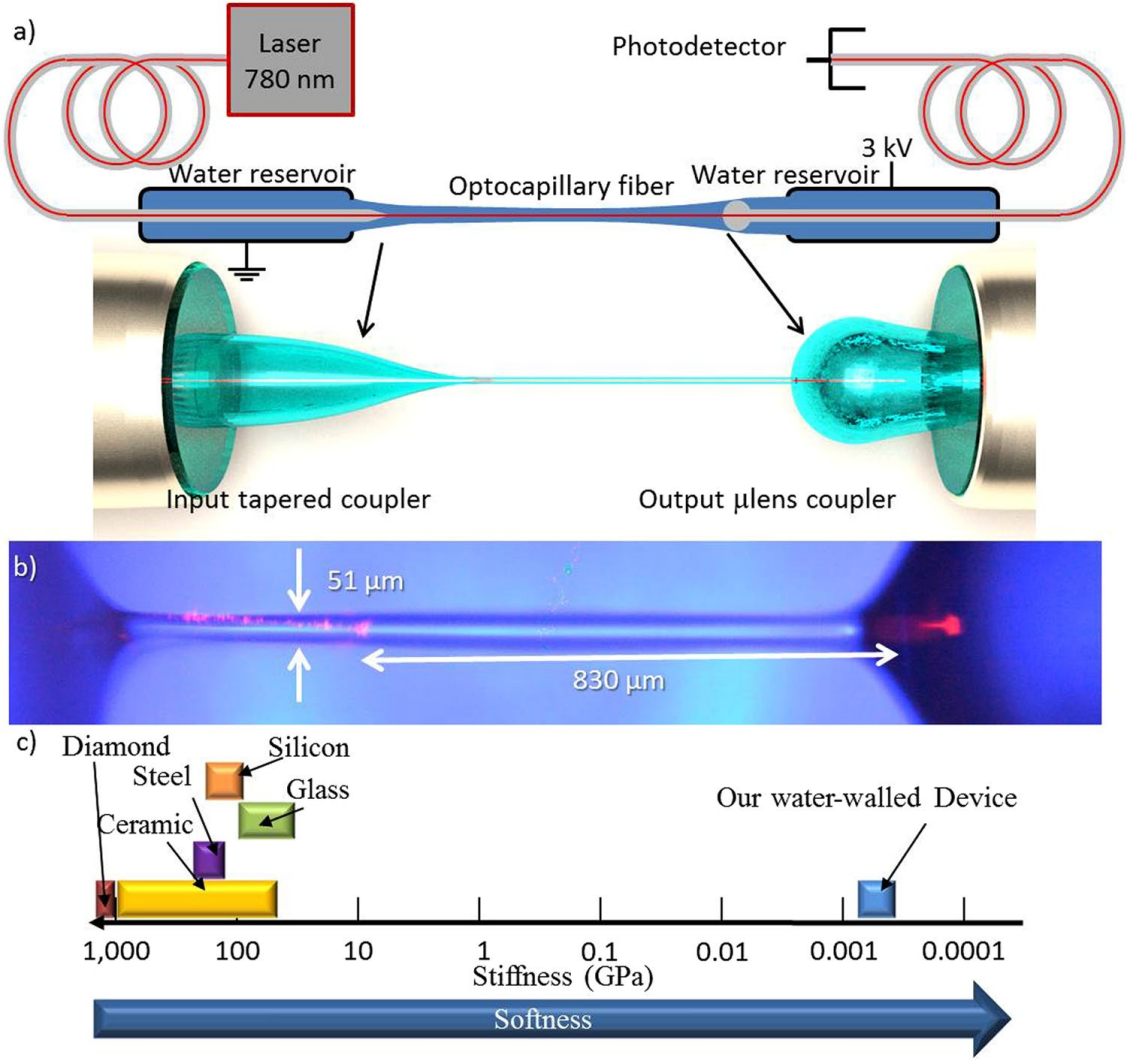
Experimental setup for water fibers: an Illustration (**a**) and a Photograph **(b**) of the water fiber setup. (**c**) Common solids softness compared with that of water-walled waveguides. The fiber length measured solid to solid from the lens surface to the tapered fiber. To improve resolution while maintaining red light contrast from the fiber, blue light was used as back illumination of the fiber. Residual red light scatterings are derived from fibers (whether in taper or lens form), and not from liquid form. Figure reproduced from our ArXiv Paper^31^.

Opto-capillary fiber fabrication^31^ is conducted by placing silica fibers into water-containing micropipette (Fig. 1a). Water is drawn from a reservoir through micropipettes in order to replenish the water bridge and to neglect evaporation effects. The hydrophilic inner part of the pipette enables rapid water flow through it to the pipette tip, while the outer hydrophobic part prevents water outflow towards the conical glass-air interface, which would maximize water-solid contact area^33,34^. The input coupler was fabricated by adiabatically tapering a silica fiber^22,35^ with a slope not exceeding ~1/20. On the other bridge base the output coupler is formed by reflowing the silica fiber tip into a fiber lens, to focus the incoming light into the fiber core^23^. Other fabrication techniques, such as applied voltage and pipette diameter, can be found on the methods section. The bridge sides are positioned in such a way that they are nearly touching each other, so that a minute fluidic contact is spontaneously created in the gap. Once the fluidic contact is established, voltage is applied between the optical couplers while the distance between the two is increased. This forms the water bridge between the optical couplers, as can be seen in Fig. 1.

We now measure the optical transmission through the water fiber by coupling in a 780 nm laser from one of its sides and measuring the transmitted power from its other side. As one can see in Fig. 1b, we measure an optical transmission larger than 54% for a 0.83 mm long water fiber.

**Figure 2:**
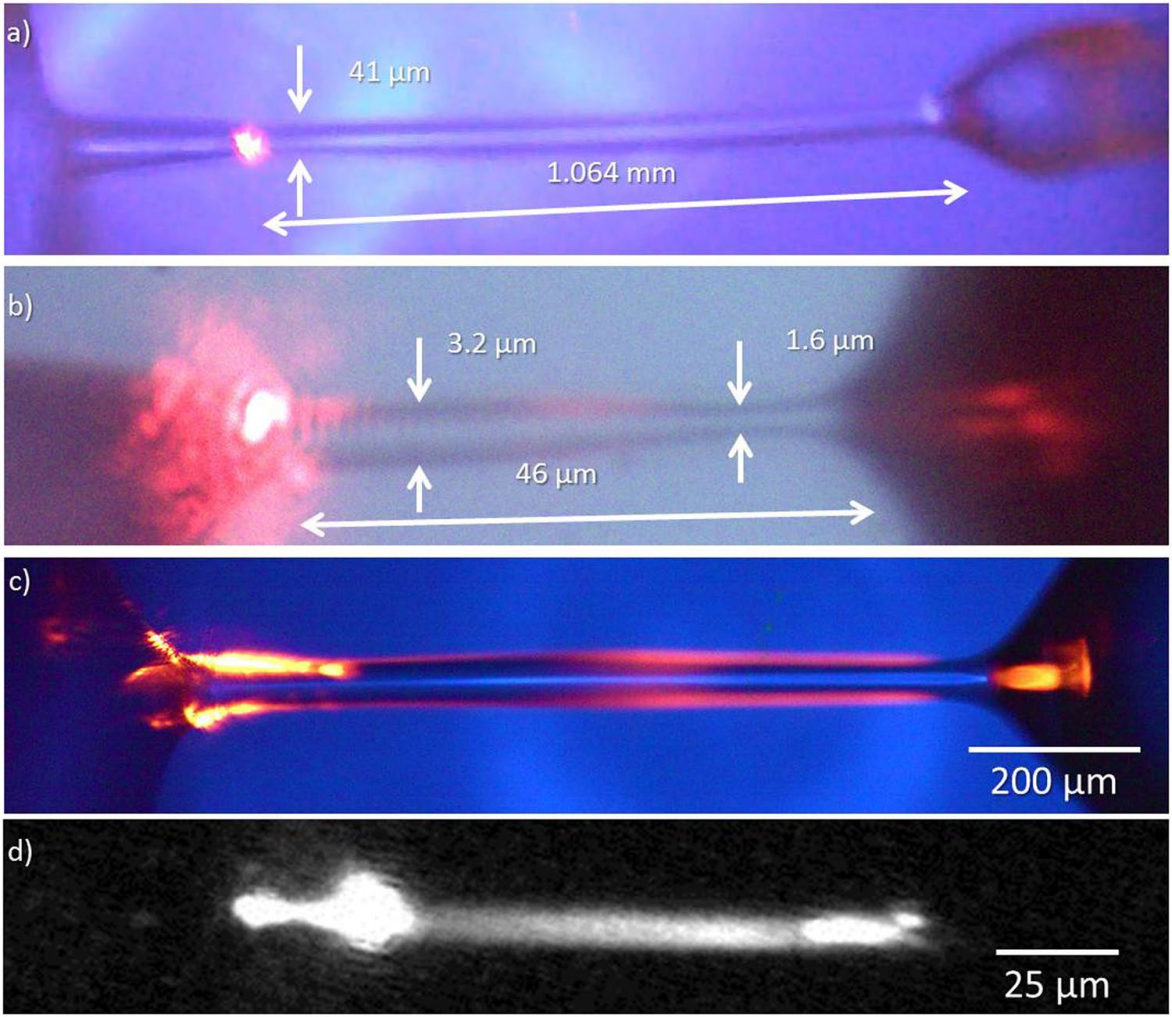
Characterizing a water fiber. (**a**) A longer than a millimeter water fiber. (**b**) a thin water fiber, (**c**) surface scattering at the water fiber liquid-phase boundary originating from capillary waves. (**d**) Fluorescent dye measurement confirming light propagation through the water fiber volume and that surface scattering in (**c**) are not due to selective propagation of light near the air-water interface. Figure reproduced from our ArXiv Paper^31^.

As for our opto-capillary water fiber geometrical characterization, they could extend over 1.06- millimeter length (Fig. 2a). The radius of the fiber could be thinner than 1.6 µm (at L = 46 µm, Fig. 2b); and at some regions, as small as 800 nm (Fig. 2b). The water fibers were maintained as long as were needed for our experiments, typically longer than 10 minutes. As one can see in Fig. 2c, light is more scattered at the fiber surface. Similar effect was studied in flat interfaces^24–27^ and originated from the scattering of light from thermal capillary waves. We had to verify that also in our case light is scattered from thermal capillary waves while mainly propagating at the internal part of the water fiber volume and not just near its surface as one might think from looking at Fig. 2c. In order to exclude the possibility, we used fluorescent dye to map light propagation. The fluorescent emission originating from the places where light passes, confirms that light, in fact, propagates in the fiber volume (Fig. 2d).

In our *experiments*, we optically interrogate the capillary oscillations of the water fiber via its effect on optical transmission. The water fiber diameter suggests that it is a highly multimode fiber. It will, therefore, act as a multimode interferometer, converting phase modulation into amplitude modulation, similar to the behavior observed when fabricating optical fiber tapers^36^. As opposed to surface capillary waves that travel near the water phase boundary (Fig. 2c and ^24–26,28,29,37^), we show oscillations of the fiber that are similar to the first mode of a guitar string. These capillary vibrations involve a transverse motion of the fiber itself, originating from internal flows^38^. Such vibrations were photographed in reference^39^. In greater detail, similar to a vibrating guitar string, the water thread vibrates at frequencies

1 $${f}_{i}=\frac{i}{2L}\sqrt{\frac{T}{{\rho }^{\text{'}}}},$$

where *ρ*′ is the string mass density per unit length, *L*; *T* is the water bridge tension and *i* = 1, 2, 3… is the index describing the order of the mode. The water fiber tension is affected by dielectric-^19^ and surface-tensions^20^; considering both terms, we get an equation for the tension according to reference^21^

2 $$T=a\cdot (\frac{\pi {D}^{2}}{8})\cdot ({\varepsilon }_{0}(\varepsilon -1){E}^{2})+b\cdot \gamma \rho D$$

where *ɛ*_0_ is the vacuum permittivity; *D* is the bridge diameter; *E* is the electric field along the bridge *ɛ* is the relative permittivity of the liquid and *ɣ* the surface tension. *a* and *b* here describe the ratio between electrostrictive-forces^19,21^ and interfacial-tension^20,21^ that are co-carrying the water bridge weight and will be fitted experimentally.

**Figure 3:**
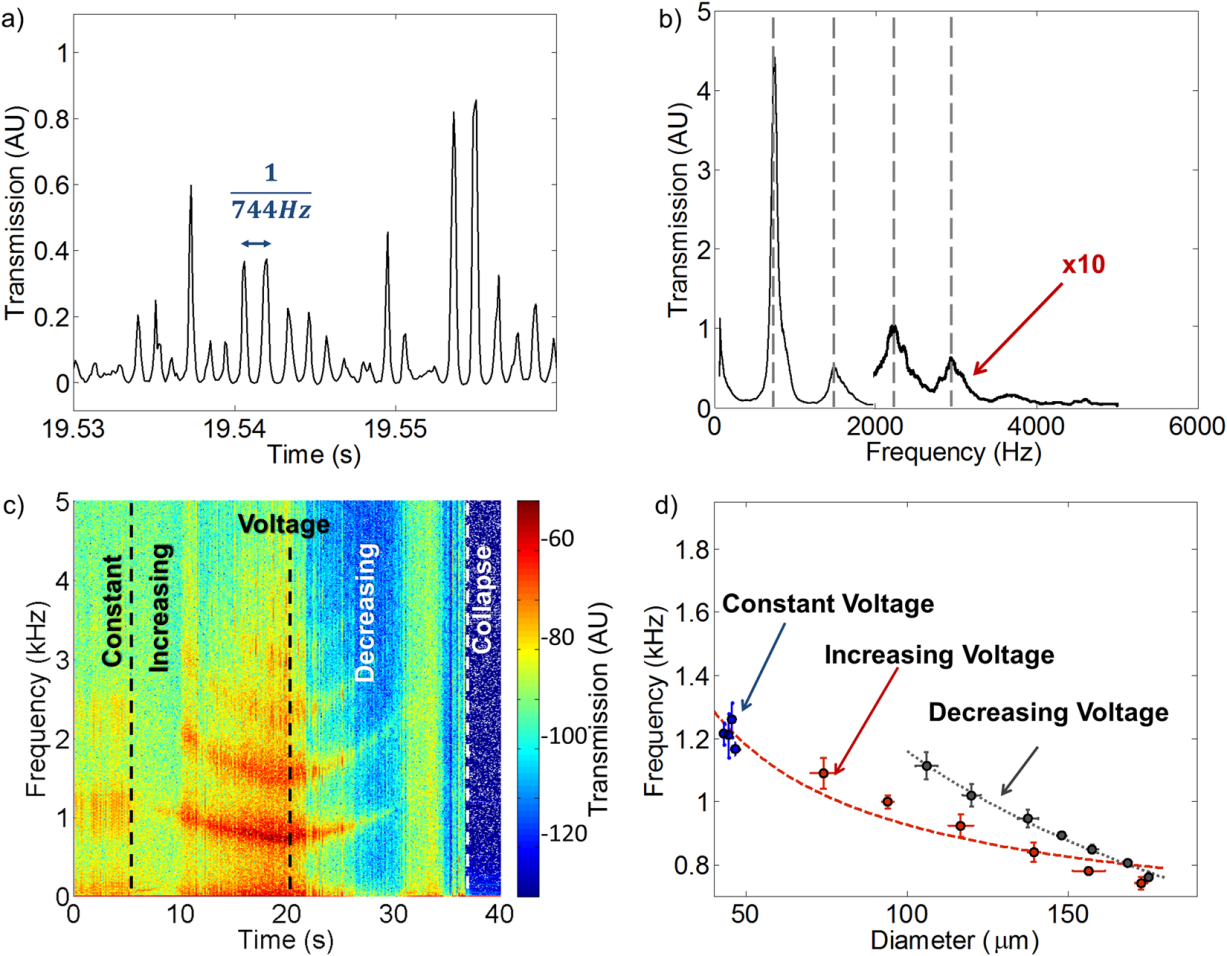
Experimentally measuring the “guitar-string” modes of the water fiber. (**a**) Time trace measurement. (**b**) Fluctuation spectrum reveals a fundamental mode and, at integer multiplications, its 3 overtones (dash lines). (**c**) Fluctuation spectrogram of a 0.94 mm long fiber, in which the voltage first is constant, then increased and finally decreased; correspondently changing the fiber diameter. Color code describes the transmission. (**d**) The fundamental frequency of the fiber, as a function of the fiber diameter (circles) together with what calculated with equations 1–2 (lines) where the ratio between dielectric and surface forces (a/b) is a free parameter. (**a**–**c**) fiber length is 0.94 mm and oscillation is optically interrogated with a photodetector (Fig. 1).

We will now optically interrogate the water fiber vibrations by their way of effect on the fiber transmission. As one can see in Fig. 3a, the transmission through the water fiber oscillates at a typical frequency of 744 Hz. This resonance frequency is also seen at the spectral domain (Fig. 3b) where a strong spectral peak is accompanied by several harmonics. While we estimate that the harmonics originates from high order fiber modes, we do not rule out the possibility of nonlinear transduction of the first order mode. We plan to resolve this dispute in the future by using a fast-enough camera. As the oscillations in the water fiber reassemble a vibrating string, it is expected that increasing the fiber diameter, D, will reduce its oscillation frequency as predicted by equations 1–2. We change the diameter of the water fiber continuously by changing the voltage to confirm this assumption (Fig. 3b), which indeed results in a chirp in the resonance frequencies as predicted by equation 1–2 (Fig. 3c and d). The frequency of the capillary oscillations is a function of the water fiber diameter and the history of this diameter. For example, and as one can see in Fig. 3 a fiber reduced to a 100-micron radius had a 20% higher frequency when compared to a fiber for which the radius was increased to 100 micron. We are still studying the origin of this hysteretic response.

**Figure 4:**
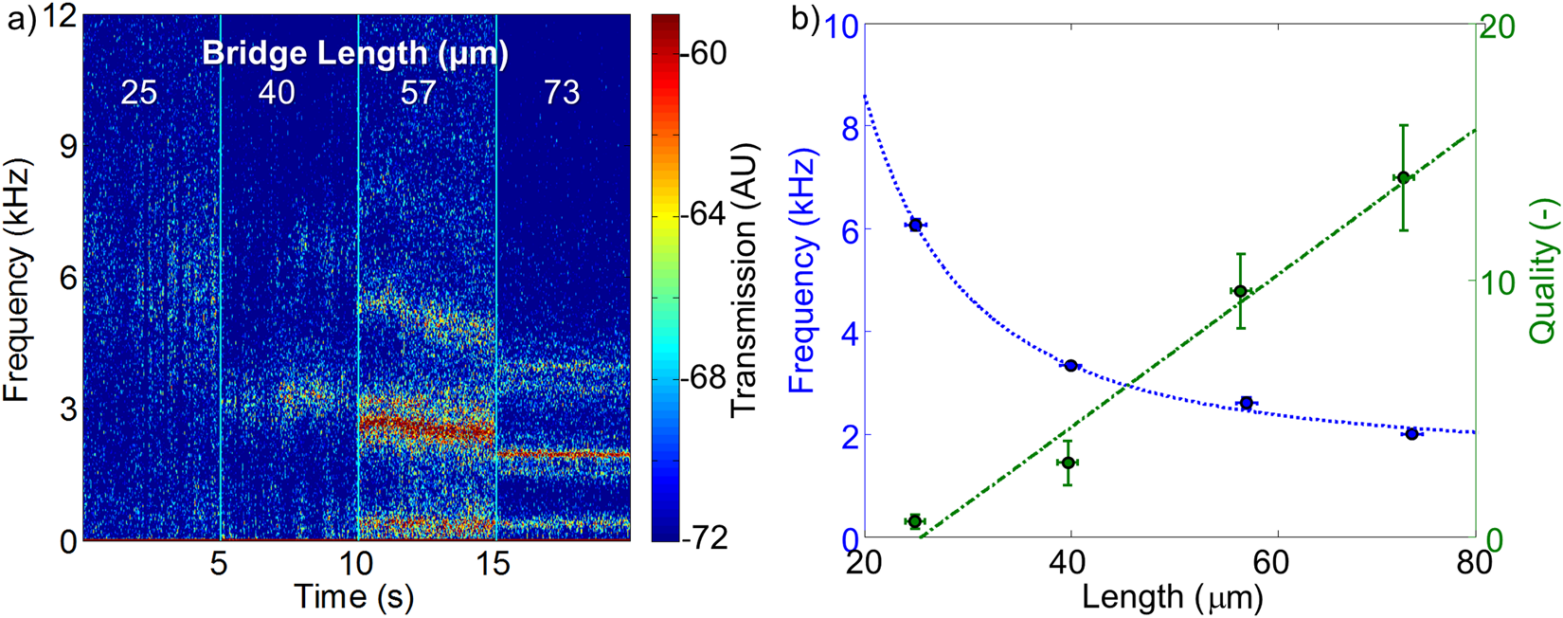
Mechanical Eigenfrequencies of the water fiber. (**a**) Spectrogram of a liquid fiber with diameter of 5.5 µm and varying length. Color code describes the transmission. (**b**) Eigenfrequency and quality factor as a function of the fiber length. The blue line shows a fit to equation (1) where *a* and *b* are the free parameter. The surface tension contribution is a magnitude of 2 greater than the dielectric tension. The green line is a guide for the eye.

In the following experiment, we monitor the oscillation frequency while changing the length of the water fiber (Fig. 4a) and keep its diameter as constant as possible (see methods, Table 1 (a). Similar to a longer guitar-string that oscillates at lower frequencies and as predicted by equations 1–2 (Fig. 4b, blue line), the measured oscillation frequency (Fig. 4b, blue circles) indeed decreases with length. The linewidth of the resonances narrows with length. Due to the viscosity of water, the energy loss is more significant at higher rates in this system. For this reason, we expect linewidth narrowing at longer fibers (oscillating at lower rates) (Fig. 4b). The capillary quality factor, Q, for our fiber is a dimensionless parameter that is inversely proportional to energy losses and linewidth. Q is defined by the ratio of stored energy to energy dissipated per oscillation cycle and is calculated to be^40^

3 $$Q=1/\alpha \lambda ,$$

4 $$\alpha =\frac{8\mu {\pi }^{2}}{\rho {\nu }_{g}{{\rm{\Lambda }}}^{2}}$$

**Table 1:** Water fiber length and radios in regard of electric potential and pipette internal diameter.

	Water fiber			Pipette’s internal diameter	
	Length [µm]	Radius [µm]	Potential [V]	Taper side [µm]	Lens side [µm]
Fig. 1b	830	51	6000	850	850
Fig. 2a	1064	20.5	6000	850	850
Fig. 2b	46	1.6–0.8	1500	150	850
Fig. 2c	820	32.5	5000	850	850
Fig. 2d	110	4.75	3000	150	150
Fig. 3	940	20–90	3000–8000	850	850
Fig. 4	24–73	2.7–3	2500	150	850

Figs 1b–4 corresponds to the illustrated water fibers through the manuscript.

and

5 $${\nu }_{g}=\sqrt{\frac{4.5\pi \gamma }{\rho {\rm{\Lambda }}},}$$

where Λ is the capillary wavelength, *α* is the absorption coefficient for capillary waves, *υ*_*g*_ is the group velocity for capillary waves, *µ* is the liquid viscosity, and *ρ* the liquid mass density. Again, as expected, *Q* decreases with length (Fig. 4, green circles), though not with the square-root behavior predicted by Eq. 3. This is probably because Eq. 3 assumes long fibers and fails for shot fibers similar to the ones that we have. The quantitative criteria for being in the underdamped regime corresponds to oscillating more than one cycle before this oscillation decays. The number of oscillations until decay is generally referred to as the quality factor, Q, of this system. As one can see in Fig. 4, the Q = 9.5 and 57 micron length fiber had one full width at half max frequency diffusion during a 5 second period while for the Q = 14 and 73 micron length fiber we could not see any frequency diffusion. The highest quality factor that we measured was 14 at 1991 Hz (Fig. 4b).

Capillary resonances were measured using optical methods, and have recorded frequencies at the audio band. Supplementary material (a) provides a soundtrack presenting the resonance convergence from white noise for a short (25 µm) water fiber to a distinct tune at a defined frequency for a long (73 µm) water fiber. As for the continuously changing water fiber diameter, presented in Supplementary material (b), the pitch is lower frequencies for increased water fiber diameter, and vice versa for decreased fiber diameter. Interestingly, this sound resembles the sound of a passing jet-plane and its related Doppler shift, as can be seen in Fig. 3b.

## Methods

The input tapered-fiber coupler is Corning HI 780 C, adiabatically tapered over a hydrogen flame as explained in ref.^22^. The output silica lensed-fiber coupler is made of a Thorlabs FTO 30 fiber that was reflowed using a CO2 laser as explained in ref.^41^.

The micropipettes are Round Borosilicate Glass Capillaries in Convenience Vials.

We use deionized water with a resistivity of 18 MΩ·cm.

During the experiment, despite the short distance and the high voltage, we do not observe an electric arc. We see such arcs only when the water fiber collapses.

Mapping light propagation in water fiber (Fig. 2d): We use a fluorescent dye that absorbs near 780 nm wavelengths and emits at 810 nm in order to map light propagation in our water fiber. We couple 780 nm light to propagate in the fiber while a side view camera is photographing the fluorescent emission, using a long pass filter. This filter transmits the fluorescent emission while it blocks the pump. The fluorescent dye is supplied by ADS LTD. and its serial number is 780 WS.

Measuring water fiber oscillation as a function of fiber diameter (Fig. 3):

We use the fact that the fiber diameter scales with voltage to control the diameter of our fiber while monitoring this diameter using a microscope. At the same time, the transmission through the fiber is monitored using a photodiode and recorded at a 10 kHz sampling rate. A Fourier transform is then performed to reveal the oscillation spectrum. The time window for the Fourier transform is 0.2 second reflecting a 5 Hz resolution and the sampling rate is about 10 times the 1 kHz oscillation rate. The audio files here were made using the Matlab command “sound” on the data grabbed as explained above.

Measuring water fiber oscillation as a function of fiber length (Fig. 4):

We change the length of the water fiber by mechanically extending the distance between its holders. While at the same time, we carefully tune the voltage in order to maintain a constant fiber diameter. The length and the diameter of the water fiber were measured using a top-view microscope. The data was recorded at a 200 kHz sampling rate. The time-window used to calculate the spectra was 0.01 seconds implying 100 Hz resolution. The audio files here were made using Matlab command “sound” on the data grabbed as explained above.

Other parameters are as appear in the following Table 1.

## Electronic supplementary material

Spectrogram of mechanical Eigenfrequencies of the water fiber with varying length

Spectrogram of mechanical Eigenfrequencies of the water fiber with varying diameter
